# Hybrid Biopolymer and Lipid Nanoparticles with Improved Transfection Efficacy for mRNA

**DOI:** 10.3390/cells9092034

**Published:** 2020-09-05

**Authors:** Christian D. Siewert, Heinrich Haas, Vera Cornet, Sara S. Nogueira, Thomas Nawroth, Lukas Uebbing, Antje Ziller, Jozef Al-Gousous, Aurel Radulescu, Martin A. Schroer, Clement E. Blanchet, Dmitri I. Svergun, Markus P. Radsak, Ugur Sahin, Peter Langguth

**Affiliations:** 1 Department of Pharmaceutics and Biopharmaceutics, Johannes Gutenberg University Mainz, D-55131 Mainz, Germany; csiewert@uni-mainz.de (C.D.S.); vcornet@uni-mainz.de (V.C.); nawroth@uni-mainz.de (T.N.); L.uebbing@uni-mainz.de (L.U.); Zillerantje@web.de (A.Z.); joalgous@uni-mainz.de (J.A.-G.); 2 BioNTech RNA Pharmaceuticals, D-55131 Mainz, Germany; Heinrich.Haas@biontech.de (H.H.); Sara.Nogueira@biontech.de (S.S.N.); sahin@uni-mainz.de (U.S.); 3 Jülich Centre for Neutron Science JCNS at Heinz Maier-Leibnitz Centrum MLZ, D-85748 Garching, Germany; a.radulescu@fz-juelich.de; 4 European Molecular Biology Laboratory EMBL Hamburg Outstation c/o Deutsches Elektronen Synchrotron DESY, 22603 Hamburg, Germany; martin.schroer@embl-hamburg.de (M.A.S.); clement.blanchet@embl-hamburg.de (C.E.B.); dmitri.svergun@embl-hamburg.de (D.I.S.); 5 IIIrd Dept. of Medicine, Johannes Gutenberg University Medical Center, Johannes Gutenberg University, D-55131 Mainz, Germany; radsak@uni-mainz.de; 6 Exp. Oncology, IIIrd Dept. of Medicine, TRON, Johannes Gutenberg University Medical Center, Johannes Gutenberg University, D-55131 Mainz, Germany

**Keywords:** vaccination, Covid-19, cancer immunotherapy, RNA, cationic polymer, cationic lipid, lipid-polymer hybrid nanoparticles, small angle scattering

## Abstract

Hybrid nanoparticles from lipidic and polymeric components were assembled to serve as vehicles for the transfection of messenger RNA (mRNA) using different portions of the cationic lipid DOTAP (1,2-Dioleoyl-3-trimethylammonium-propane) and the cationic biopolymer protamine as model systems. Two different sequential assembly approaches in comparison with a direct single-step protocol were applied, and molecular organization in correlation with biological activity of the resulting nanoparticle systems was investigated. Differences in the structure of the nanoparticles were revealed by thorough physicochemical characterization including small angle neutron scattering (SANS), small angle X-ray scattering (SAXS), and cryogenic transmission electron microscopy (cryo-TEM). All hybrid systems, combining lipid and polymer, displayed significantly increased transfection in comparison to lipid/mRNA and polymer/mRNA particles alone. For the hybrid nanoparticles, characteristic differences regarding the internal organization, release characteristics, and activity were determined depending on the assembly route. The systems with the highest transfection efficacy were characterized by a heterogenous internal organization, accompanied by facilitated release. Such a system could be best obtained by the single step protocol, starting with a lipid and polymer mixture for nanoparticle formation.

## 1. Introduction

Messenger RNA (mRNA)-based pharmaceuticals represent a new class of therapeutics for versatile medical applications [1,2,3,4]. mRNA can be used to encode functional peptides, such as tumor-associated antigens for cancer immunotherapy or antigens for vaccination against infectious diseases. Its limitation is due to its instability, fast degradation via ribonuclease (RNase) enzymes and low cellular uptake [5,6]. Therefore, suitable delivery systems need to be developed to increase the uptake of mRNA and make it applicable to patients [7,8,9,10,11]. Most approaches for RNA delivery are based on nanoparticles comprising either cationic lipids or polymers for complexation and condensation of the RNA [9,12,13]. Nanoparticles from cationic or ionizable lipids are among the most established systems for clinical application of nucleic acid delivery [14,15], and different types of lipid-based RNA nanoparticle products have been successfully brought into clinical development [4,15] or into clinical practice [16]. However, for many applications, further improved, tailored vehicles are required to account for the specific necessities for RNA delivery towards a given cell type or for a certain therapeutic intervention. For example, RNA delivery to blood or immune cells would open a scope of new therapeutic options in cancer (immune)therapy and in various other indications, for example, in the field of inflammatory diseases. One approach for assembly of advanced, next-generation delivery systems is based on the combination of polycationic polymers together with positively charged lipids, which combines the advantageous physical properties of polymers and lipids in the nanoparticles [17,18]. Different lipid–polymer-hybrid nanoparticle production methods have been reported, wherein single and multiple step protocols, predominantly using nanoprecipitation- or solvent evaporation-based manufacturing routes, have been applied [6,17,18,19,20]. Many of those approaches aimed to produce particles with a core-shell organization, as this was expected to be favourable for biological activity [17,18,19,20,21]. Here, we systematically investigated different manufacturing protocols for the assembly of hybrid polymer and lipid nanoparticles and correlated physicochemical, in particular structural, characteristics with biological activity in vitro and in vivo. We used three manufacturing sequences each including dual asymmetric centrifugation (DAC) to investigate the change in structural properties and discovered that they can lead to different structures than have been shown for other hybrid particles in the past.

In order to facilitate universally valid conclusions, well-known complexation agents were chosen. Protamine was used as the model biopolymer and complexation agent. It is a naturally occurring protein containing more than two-thirds of positively charged L-arginine and is known to stabilize DNA during spermatogenesis. Due to the high amount of cationic L-arginine, protamine has the ability to complex mRNA and protect it from enzymatic RNase degradation [22]. Protamine/mRNA complexes can act as danger signals, activate white blood cells, and are generally able to stimulate immune responses [23]. As the cationic lipid, dioleoyl-3-trimethylammonium propane (DOTAP) was selected, one of the longest known and best characterized lipids for mRNA cell transfection [10,24].

The combination of protamine and DOTAP was used before, e.g., in the systemic gene therapy of the E1A Gene and Herpes Simplex Virus 1 Thymidine Kinase [25,26,27]. The effect of the combination of both seemed to be advantageous in the delivery of DNA and siRNA [28,29,30,31,32,33]. In this work, lipid nanoparticles comprising mRNA were manufactured at various DOTAP:protamine ratios, using different assembly routes. Two different sequential assembly approaches starting with a core comprising either one of the complexing agents were compared with a single-step self-assembly protocol for nanoparticle formation.

Thorough physicochemical and biological characterization of these systems was performed. Dynamic and electrophoretic light scattering (DLS/ELS), as well as small angle neutron scattering (SANS), small angle X-ray scattering (SAXS), and cryogenic Transmission Electron Microscopy (cryo-TEM) was used to reveal the structural information of the particles. Cryo-TEM allows direct insight into the morphology of nanoparticles; however there is a higher possibility of misleading artefacts [34]. Small angle scattering measurements, which were performed in parallel, are a powerful method for investigation of biomembranes [35,36,37], as well as colloidal systems such as polyplex formulations [38,39,40,41] and lipoplexes [42,43,44,45,46]. In previous studies the formation of a condensed lamellar structure was identified by interaction between negatively charged DNA/RNA and positively charged DOTAP [39,42,44]. Scattering with neutrons (SANS) additionally enables us to distinguish between material domains by generating scattering profiles with complementary contrast conditions by hydrogen/deuterium contrast variation [47,48,49,50,51,52]. The structural characteristics inside the formulations were observed in comparison to their biological activity, as investigated in vitro in HEK 293 and C2C12 cells by luciferase assay and in vivo in mice by bioluminescence imaging. The results allowed proposing a model for the correlation between the structural and functional characteristics of the different products.

## 2. Materials and Methods

### 2.1. Materials

Protamine sulfate salt from salmon Grade X (Mol. W. approx. 5.1 kDa, solubility in H_2_O: 10 mg/mL, lot: #SLBC1855V) was purchased from Sigma Aldrich (St. Louis, MO, USA). Anhydrous D(+)-Glucose (lot: K46419537525) and DOTAP (Mol. W. 698.5 g/mol, lot: 890890C-25MG-A-165) was purchased from Merck KGaA (Darmstadt, Germany). BioScience-Grade 2-[4-(2-hydroxyethyl) piperazin-1-yl] ethane sulfonic acid (HEPES) (lot: 294215827) and nuclease-free water were purchased from Carl Roth (Karlsruhe, Germany). Luciferase-coding mRNA with about ~2000 bases was synthesized by internal protocols at BioNTech (Mainz, Germany) (lot: 12-56-20, EH180214-01) [53,54]. Heparin (lot: Y0001282 Batch 2.1) was purchased from Sigma Aldrich (St. Louis, MO, USA). Silica beads of 0.5 mm size and 0.5–2 mL skirted TwistTop vials from Sorenson BioScience Inc (Salt Lake City, UT, USA) were used for nanoparticle formation. For quantitative mRNA determination, the Quant-iT RiboGreen assay kit (lot: 1709963) from Thermo analytics (Calumet, MI, USA) was used and samples were measured in 96-well plates with a 190 µm bottom thickness using a TECAN infinite F200 plate reader (Tecan Trading AG, Männerdorf, Switzerland) with an excitation wavelength of 465 nm and an emission wavelength of 535 nm. Fetal bovine serum (FBS) was purchased from Sigma Aldrich (St. Louis, MO, USA).

### 2.2. Methods

#### 2.2.1. Preparation of Lipid Films

DOTAP films were produced from a stock solution (25 mg/mL in chloroform) in twist top vials (0.65 mL). Chloroform was evaporated using a Speed vac SVC 200 (Thermo Fisher Scientific Inc., Wlatham, MA, USA) for 1 h at 1 mbar. The lipid films were frozen overnight at −14 °C.

#### 2.2.2. Preparation of Particles

Stock solutions were prepared for luciferase-coding mRNA (2.45 mg/mL) and protamine (8 mg/mL) in HEPES-buffered glucose (20 mM HEPES with 5% (*w*/*v*) D-(+)-Glucose) at pH 7.0–7.2 and 300 ± 20 mOsmol. The concentration of protamine and DOTAP varied according to the desired proportions of positive charges derived from protamine: 15% (LOW), 45%/55% (MID), and 85% (HIGH). Three different assembly routes were chosen, either starting with a negatively charged polymer/mRNA core and then adding a cationic lipid (polymer core, PC) or vice versa, starting with a negatively charged lipid/mRNA core and then adding a cationic polymer (polymer shell, PS). In the third assembly method, first the lipid and polymer were mixed, and with this mixture, particles with mRNA were formed in a single-step protocol (mixed particles, MP). Particles were prepared using a Phoenix RS-VA10 Vortexer (Phoenix Instrument, Garbsen, Germany) (10 s, 25 W) and a SpeedMixer^TM^ DAC 150.1 CM 41 (Hauschild &Co KG, Hamm, Germany) (5 min, 3000 rpm). The exact concentrations, charge-, and weight ratios of the particles are given in Table S1 (Supplementary Materials). It should be noted that the ratio of PS_MID_ slightly differed from the ratio of PC_MID_ and MP_MID_—this was necessary in order to obtain negatively charged intermediate particles and to avoid aggregation during manufacturing.

#### 2.2.3. Particle Size and Zeta Potential Measurement

Particle sizes and zeta potentials were determined using dynamic and electrophoretic light scattering (Malvern Zetasizer Nano-zs, Malvern, UK) in Y195.1 cuvettes DTS1070 (size) and DTS1070 cuvettes (zeta potential). The refractive index was set to 1.590, and nanoparticles were diluted to 0.05–0.1 mg/mL final mRNA concentration in 5 mM NaCl solution and measured at 25 °C. For size determination, a backscatter detection at an angle of 173° was used. Particle sizes were obtained as the Z_average_ [nm] by fitting the correlation function using the cumulate method. The zeta potential was measured at a concentration of 0.01–0.02 mg/mL mRNA concentration in 5 mM NaCl at 25 °C. The number for the measurements for each sample system was between *n* = 5 (low/high concentrated protamine boundary conditions) and *n* = 12 (moderately concentrated protamine conditions).

#### 2.2.4. Accessible mRNA Concentration

For evaluating the amount of accessible mRNA within the particles, the Quant-iT RiboGreen RNA reagent kit from Invitrogen (Carlsbad, CA, USA) was used. The experimental procedure was performed according to the manufacturer’s guideline. For analysis, a Tecan plate reader Infinite 200 from Tecan Trading AG (Männerdorf, Switzerland) was used at an excitation wavelength of 465 nm and an emission wavelength of 535 nm. The test was performed in 1x Tris-EDTA (TE)-buffer (diluted from Roti^®^-Stock 100× TE buffer, BioScience-Grade, obtained from Carl Roth GmbH + Co. KG (Karlsruhe, Germany)). All results were obtained from at least three independent samples, measured in triplicate.

#### 2.2.5. mRNA Release Assay in the Presence of Heparin

Particles containing 0.1 mg/mL mRNA were incubated at 37 °C for 20 min. Subsequently, heparin solution (heparin 5–50 mg/mL in 500 mM NaCl) was added to the mixture with a 0-, 12.5-, 25-, 50- or 100-fold molar ratio (with regard to mRNA) at pH 7–7.5. The samples were then again incubated at 37 °C for 20 min. As a control, an equal volume of HEPES-buffered glucose (HBG) buffer (5% (*w*/*v*) glucose, 10 mM HEPES at pH 7.1) was used to replace the heparin solution. The released mRNA concentration was quantified using the Quant-iT RiboGreen assay as described above, and a Weibull equation was used to describe the free mRNA concentration (Berkeley Madonna v9.1, University of California, Department of Molecular and Cellular Biology, Berkeley; United States) in the presence of different heparin concentrations.

(1) $$c(mRNA_{free})\left[\%\right]=mRNA_{0}+Q\times (1-e^{-{\left(\frac{c\left(Hep\right)}{a}\right)}^{b}})$$

where *mRNA_0_* is the free mRNA concentration before adding heparin, *a* is the scale factor, *b* is the shape factor, and *Q* is the percentage of free mRNA released from the particles at an infinitely high heparin concentration.

#### 2.2.6. Small Angle Neutron Scattering

SANS measurements were conducted using the KWS2 instrument at the FRM-II reactor (JCNS outstation at the MLZ, Garching, Germany) [55]. The scattering profiles *I(q)* were expressed as *q* (scattering vector, aka momentum transfer, Equation (2) [56], with half-scattering angle *θ* and neutron wavelength *λ_n_* = 6 Å. The common q-range was 0.0015—0.5 Å^−1^.

(2) $$q=\frac{4\pi }{\lambda }\sin\theta$$

Three sample-to-detector distances of 2, 8, and 20 m were used. The neutron beam size at the detector was 6 × 8 mm^2^. Samples with D_2_O concentrations below 33% were measured in quartz cells with 1 mm path length, and samples with higher D_2_O concentrations (≥ 33%) in quartz cells with 2 mm path length. Particles were measured in HBG buffer at pH 7.2. Incoherent scattering, buffer scattering, and the empty cell scattering were subtracted before merging the data from three sample-to-detector distances. Further data reduction steps, such as intensity scaling and radial integration, were performed with QTIplot 1.0.0-rc3, Qt version 4.8.7 (IONDEV SRL, Bucharest, Romania) and Origin^®^ 2016 (OriginLab, Northampton, MA, USA). The slope of the scattering curves at moderate q-values (q × d > 1, with d the average particle size) provides the Porod exponent, P, indicating differences in the morphology of the particle topology regarding the molecular fractals [57,58,59,60]. Exponents with P = 4 (*I(q)* ∝ *q*^−4^) indicate superstructures with high globularity and smooth surfaces, while P = 3 indicates rough surfaces or collapsed polymer chains [61]. In particular, mass-fractal and surface-fractal interfaces exhibit primarily power-law scattering with 1 < P < 3 and 3 < P < 4, respectively.

Different contrasts were obtained by varying the H_2_O to D_2_O composition of the buffer. The contrast matching point was determined by obtaining the x-intercept of a plot of √I0 vs.% D_2_O in buffer (see Supplementary Data). These intersections indicate medium conditions for which the particles are ‘contrast-matched’ and do not contribute to the scattering of a sample [35,51,57]. The D_2_O matching points were observed for pure mRNA, protamine, and DOTAP, as well as for the hybrid nanoparticles and were compared to scattering length densities from the literature [35,62].

#### 2.2.7. Small Angle X-ray Scattering

SAXS measurements were conducted using the P12 beamline operated by EMBL at the PETRA III synchrotron X-ray source at DESY (Hamburg, Germany). The beamline is especially suitable for biological samples in solution with its high intensity of 10^13^ ph/s and highly focused beam [63]. Nanoparticles in HBG buffer at pH 7.2 were injected into a glass capillary mounted within an air gap between two vacuum sealing Kapton windows, and monochromatic X-rays of a wavelength of 0.124 nm (10 keV X-ray energy) were scattered at the sample with an exposure time of 0.5 s. The detector Pilatus 6 M (Dectris Ltd., Baden-Daettwil, Switzerland) was adjusted within a distance of 3.0 m, resulting in a q-range of 0.003–0.73 Å^−1^. The beam size at the detector was 0.2 × 0.12 mm^2^. Radial averaging of the two-dimensional scattering patterns was done by ATSAS SASFLOW software [64]. For buffer subtraction, ATSAS PRIMUS [65] was used, and the determination of the peak area and height was done with the ATSAS program PEAK [65]. Further data illustration was done with QTI Plot 1.0.0-rc9, Qt version 5.12.2, Copyright 2004–2019 Ion Vasilief (IONDEV SRL, Bucharest, Romania).

#### 2.2.8. Cryo-TEM Images

Grids were hydrophilized by oxygen plasma (negative surface charge), and each sample was vortexed for 30 s before grid preparation. The samples were preserved in vitrified ice supported by QuantiFoil^®^ R2/1 holey carbon films from Quantifoil Micro Tools GmbH (Jena, Germany): 6 µL sample suspension was applied and immediately followed by vitrification in liquid ethane at −180 °C with a Leica EM GP from Leica Microsystems GmbH (Wetzlar, Germany). The final grids were stored under liquid nitrogen. Cryogenic TEM imaging was performed with a Zeiss Libra^®^ 120 from Carl Zeiss (Jena, Germany) at 120 kV acceleration voltage under liquid N2 cryo-conditions, and the images were taken with a Gatan UltraScan^®^ ccd camera (Gatan, Inc., Pleasanton, CA, USA). Vitreous ice grids were transferred into the electron microscope using a cryostage that maintains the grids at a temperature below −170 °C. Images of each grid were acquired at multiple scales to assess the overall distribution of the specimen.

#### 2.2.9. In Vitro Model: Cell Preparation, Cellular Uptake

HeK293 and C2C12 cells were cultured in DMEM (Gibco Life technologies, Carlsbad, CA, USA) with 10% FBS at 5000 and 20,000 cells/well on a 96 well plate. Particles were diluted into medium to 5–10 μg/mL of luciferase-encoding mRNA. The translation product of lucRNA was detected by the luciferase assay (Bright-Glo Luciferase kit, Promega, Madison, WI, USA) performed 24 h post-treatment. Additionally, the viability of transfected cells was evaluated by using the cell proliferation Kit II (XTT, from Sigma Aldrich, St. Louis, MO, USA) after 4 h of incubation with XTT reagent according to its specifications. All measurements were performed using a TECAN reader Infinite m200PRO (Tecan Trading AG, Männerdorf, Switzerland).

#### 2.2.10. In Vivo Experiments

BALB/c_Rj mice (8 weeks/female) were obtained from the central animal house, Zentrale Versuchstiereinrichtung (ZVTE), of the Johannes Gutenberg University Mainz (License: G18-12-007). Group size was 3 per test item and 2 for negative control (buffer). The total number of test groups was 7. The mice were anaesthetized with isoflurane; the dose range was 4 µg RNA per mouse injected intramuscular (i.m.) in a volume of 20 µL into the right and left hind legs. Biological activity of luciferase-encoding mRNA was evaluated by dorsal bioluminescence imaging using an IVIS spectrum imaging system (Caliper Life Sciences, Alameda, CA, USA) Bin 4 (1 min) and Bin 8 (1 min). Readout was performed after 0 h, 6 h, 24 h, and 48 h. Bioluminescence was presented as colour-scaled images superimposed on grayscale photos. Quantification resulted from the average radiance (quantified as photons/sec/cm^2^/sr) from each pixel inside the regions of interest (ROIs).

#### 2.2.11. Statistical Analysis

Two-way analysis of variance (ANOVA) was performed for in vitro analysis, when both protamine concentration and particle topologies were compared, with replication followed by the Bonferroni multiple comparison test using the data analysis tools in MS Excel^®^ 2016 (Microsoft, Redmond, Wa, USA). For in vivo analysis, one-way ANOVA was performed followed by Tukey’s multiple comparisons test using GraphPad Prism 6 (GraphPad Software, Jolla, CA, USA). Data are shown as the mean ± S.D. for each experiment. *p*-values < 0.05 were considered significant. Other significance levels are marked as ** for *p* < 0.01 and *** for *p* < 0.001.

## 3. Results

### 3.1. Preparation of Particles at A Given Mixing Ratio and Different Assembly Routes

**Figure 1:**
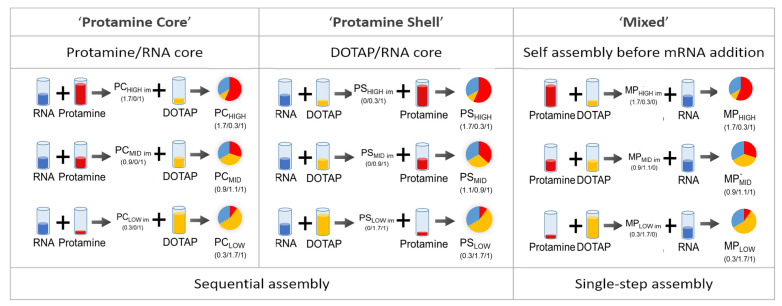
Graphical representation of the concept of different assembly routes for the preparation of hybrid particles. Three different particle topologies with protamine as either the first added component (PC), the last added component (PS), or in a mixture with DOTAP before RNA addition (MP) were compared. The N/P ratio composition is given underneath the pie charts (protamine/DOTAP/mRNA).

The ratio between positively charged nitrogen (N) in the excipients and negatively charged phosphorus (P) in mRNA is decisive for physicochemical particle characteristics and biological activity [4,15,40,66]. The N/P ratio as given here was obtained through calculating the weight carrying 1 mol unit charge. Luciferase-coding mRNA has approx. 2000 bases with a 340.5 g/mol negative charge. Therefore, 1 g mRNA is equivalent to 2.94 mmol negative charge. At physiological pH, the phosphate groups are anionic, based on the relevant pKa of the phosphate groups in the RNA backbone [67]. The amount of positive charges in protamine was determined according to the sulfate concentration given by the manufacturer. Protamine has 3.6 mmol positive charges per gram of substance. Potentiometric titration (supplement) indicates that 0.35 mmol are unionized at pH 7.2 (manufacturing conditions). DOTAP has a molecular weight of 698.5 g/mol and one quaternary amine group per molecule. Therefore, it is ionized independently of the pH value. The amount of positive charges per gram DOTAP is 1.43 mmol positive charge. In summary, 1 g substance contains 2.94 mmol negative charge (RNA), 3.6 mmol positive charge (protamine), and 1.43 mmol positive charge (DOTAP). Three different assembly routes were applied, two consisting of sequential addition of the polymer and lipid to the RNA, and a single-step protocol where a polymer/lipid mixture was added. More specifically, the preparation protocols consisted of (1) the formation of an mRNA/polymer core (PC) to which lipid was added in the second step, (2) the formation of a mRNA/lipid core to which polymer as a shell was added (PS), (3) the formation of a lipid/polymer mixture, which was added to the mRNA to form mixed particles (MP). All particles were manufactured at a final N/P ratio of 2. For each assembly route, three different N/P ratio compositions of protamine and DOTAP were manufactured. The N/P ratio composition (protamine/DOTAP/mRNA) for the respective particles is given in the graphical illustration below (Figure 1). For the 0.3/1 intermediates (PC_LOW_, PS_HIGH_), the mRNA was only partially bound to the cationic vehicle. Therefore, for the final PC_LOW_ and PS_HIGH_ particles, interactions with the second added complexing agent with both pre-formed particles and free RNA have to be taken into account, which may lead to particle compositions with higher complexity.

### 3.2. Physicochemical Characterization and Heparin-Driven mRNA Release Assay

The results of the DLS/ELS measurements are summarized in Table 1 and Table S2. Discrete particles with a confined size (Z_ave_ < 300 nm, PDI < 0.3) were obtained irrespective of the assembly route and the protamine:DOTAP ratio at the selected final N/P ratio of 2. Under these conditions, all particles had a positive zeta potential >20 mV. With increasing amount of protamine (_LOW_>_MID_>_HIGH_), there was a tendency that the MP particles decreased in size, whereas PS particles increased in size, and PC particles did not change significantly.

**Figure 2:**
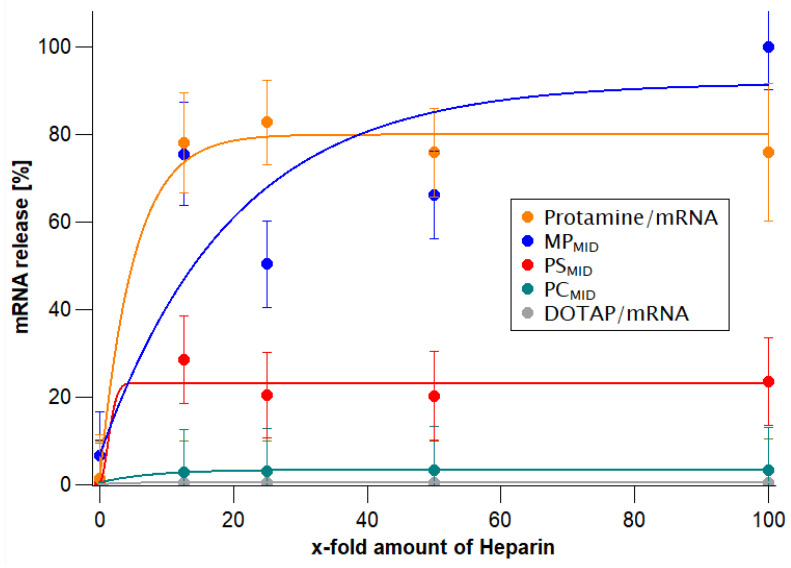
Release of mRNA after the addition of heparin in H_2_O, tested at an N/P ratio of 2 for protamine/mRNA particles (orange), DOTAP/mRNA particles (grey), MP_MID_ (blue), PC_MID_ (green), and PS_MID_ (red) particles. The curves are drawn to guide the eye.

To evaluate how much mRNA could be enclosed in the particles, the amount of mRNA accessible to a fluorescent dye was taken as an indirect parameter showing how much unbound or superficially bound mRNA there was. All particles showed a low mRNA accessibility. However, twice as much accessible mRNA was measured for the particles that were produced with the single-step protocol (MP) in comparison to the other assembly routes (PS/PC). To differentiate the particle systems further, the heparin-triggered mRNA release was investigated (Figure 2). Anionic heparin interacts with the positive charges from DOTAP and protamine, thus heparin is competing with anionic mRNA at the interacting sites, leading to a release of mRNA from the cationic complexing agents. The added heparin concentration was increased from a 10-fold to a 100-fold excess compared to the mRNA concentration (%*w*/*w*). Particles with an N/P ratio of 2 consisting of only DOTAP/mRNA or protamine/mRNA were measured in comparison to hybrid particles that had comparable DOTAP/protamine/mRNA absolute ratios but were obtained from different assembly routes. For DOTAP/mRNA lipoplexes, even a 100-fold excess of heparin led to no observable mRNA release, whereas for protamine/mRNA polyplexes, the 10-fold excess was sufficient to release approx. 80% of the bound mRNA. The release of mRNA from the hybrid particles depended clearly on the assembly routes. Whereas PC_MID_ particles showed negligible release of mRNA, for PS_MID,_ moderate mRNA release was observed, and for MP_MID_ particles, the lowest amount of heparin was required for mRNA release. The high mRNA release from MP_MID_ particles is in accordance with the higher amount of accessible mRNA in the Quant-iT RiboGreen assay (Table 1).

### 3.3. Advanced Structure Investigation by SANS, SAXS, and Cryo-TEM

**Figure 3:**
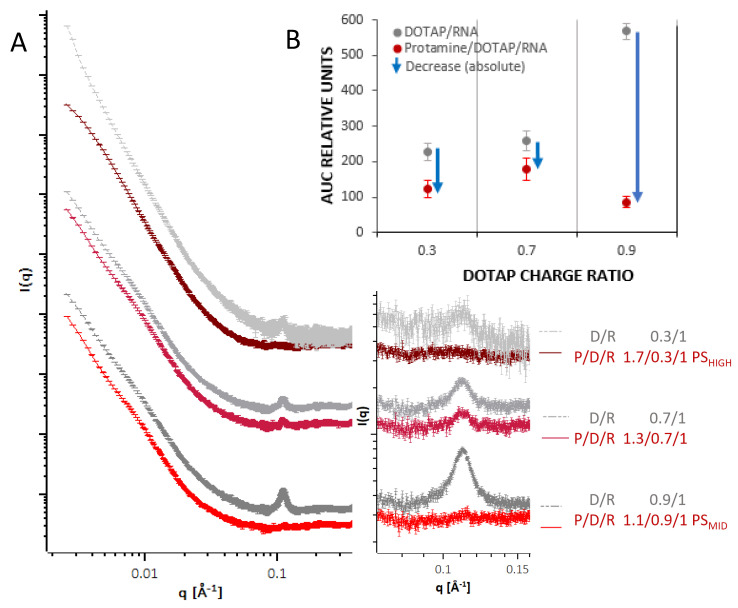
(**A**) Double logarithmic plot of SAXS profiles of negatively charged PS particle intermediates (DOTAP/RNA, grey curves) of three different charge ratios of DOTAP (0.3, 0.7, 0.9) with a constant charge ratio of RNA (1.0) and for each curve below the corresponding curve after protamine addition to form the final PS particle (red curves) in HBG buffer (pH 7.2). Colour darkening indicates a higher proportion of protamine in the particle for the red curves and of DOTAP in the grey curves. The magnification shows the q-range around 0.11 Å^−1^ where the peak is located. Curves were shifted vertically for clarity. (**B**) Area under the curve (AUC) for the three different N/P ratios. The blue arrow indicates the absolute reduction of the AUCs after protamine addition.

In addition to physicochemical characterization by classical laboratory methods, the structure and molecular organization of the delivery systems as assembled by the different protocols was investigated by a combination of SAXS and SANS measurements, and the morphology was revealed by cryo-TEM imaging. In Figure 3, results from SAXS measurements from the different systems are summarized. For formulations where the RNA was complexed with DOTAP (PS intermediates) to form a negatively charged intermediate, a Bragg peak at around q ~0.1 A^−1^ (corresponding to a repeat distance of approx. 63 Å) was measured (Figure 3A, grey curves). The peak area increased monotonously with the fraction of DOTAP relative to the RNA (0.3, 0.7, 0.9). The peak characteristics are in accordance with measurements from comparable systems of cationic liposome DNA and RNA complexes in previous studies [39,42,44,45,46]. The pattern is thought to emerge from repeating lipid bilayers, where the RNA is present in the hydrophilic slabs, intercalated between the lipid head groups of adjacent bilayers.

When protamine was added to these complexes (PS particles, red curves), the peaks from these DOTAP/RNA lipoplexes decreased or almost vanished. Except for the different area of the Bragg peak, before and after having added the protamine, all curves demonstrated a similar overall shape, characterized by a smooth decay of the intensity as a function of q, without further prominent modulations in gradient for q < 0.03 A^−1^. This indicates, that upon protamine addition, the internal organization was changing while the overall structure was largely unaffected. Figure 3B shows the total reduction of the area of the Bragg peak upon protamine addition for each N/P ratio. As a trend, the peak area was reduced more for a higher initial peak area, but to a similar (low) level in the final state. It appears that the protamine competed with DOTAP for RNA binding, and a certain fraction of the organized RNA lipid complexes was disassociated due to the interaction with protamine.

**Figure 4:**
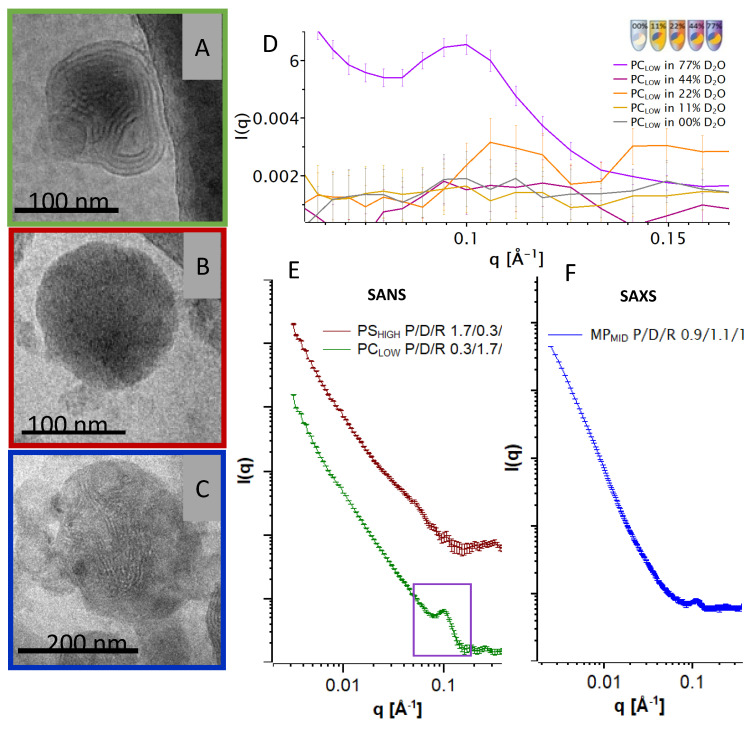
(**A**–**C**) Cryo-TEM pictures of the respective particles shown in the same colour as the corresponding curves. (**D**) Intensity scattering of PC_LOW_ particles in a q-range of 0.03–0.18 Å^−1^ at different D_2_O concentrations. The peak at 0.1 Å^−1^ is present when the scattering length density of the buffer diverges from the scattering length density of the lipid. (**E**) Double logarithmic plot of SANS scattering of PC_LOW_ (N/P 0.3/1.7/1) and PS_HIGH_ (N/P 1.7/0.3/1) at 77% D_2_O. Curves were shifted vertically for clarity. (**F**) Double logarithmic plot of the SAXS curve of MP_MID_.

The cryo-TEM pictures of the final particles with originally negatively charged intermediates: PS_HIGH_, PC_LOW,_ and MP_MID,_ are shown in Figure 4. The PC_LOW_ (protamine poor) image (A) shows compact particles with continuous and multilamellar internal structure. Some particles also showed discontinuous multilamellar organization with an electron-dense core. Picture (B) shows that the PS_HIGH_ (protamine rich) particles are also compact and round in shape but with a more homogenous structure without visible lamellar organization in accordance with homogenous, electron-dense particles (not shown). In comparison, the cryo-TEM image of the single-step assembled particles MP_MID_ in Figure 4C shows a large nanoparticle with more discontinuous lamellar patterns embedded in electron-dense areas within the particles. The corresponding SAXS curve (F) shows a Bragg peak at the same q- position as seen in Figure 3.

The systems from which the cryo-TEM images were generated were further investigated by SAXS/SANS measurements. SAXS experiments of PS_HIGH_ and PC_LOW_ resulted in weak peaks for both particles (not shown). SANS scattering curves revealed a clearer picture thanks to the possibility of adjusting the scattering density of the buffer to match the scattering density from a single component, making it possible to make these contributions to the particle ‘transparent’ to neutrons. (For detailed information on the contrast matching see Supplementary Data Figures S1–S7 and Table S3). Therefore, the individual scattering contributions from each component can be visualized. In Figure 4D, the scattering curves of PC_LOW_ at different D_2_O ratios are depicted in detail at q-values between 0.06 and 0.16 Å^−1^. The peak is only observable at high D_2_O concentrations, which account for the high neutron scattering-length density (NSLD) of the bulk phase, therefore indicating that this reflection derives mainly from a component with a low neutron scattering-length density (NSLD) such as the hydrocarbon moieties of the ordered DOTAP bilayers. Figure 4E shows the corresponding SANS pattern of the PC_LOW_ (protamine poor) particles with a peak at a q-value of ~0.1 Å^−1^. When following the PS_HIGH_ protocol (protamine rich), this peak is barely present. The two scattering curves follow a power law of l(q) ∝ q-P with exponents of 2 < P < 3.5 indicating that the particles exhibit an anisotropic or fractal structure that may be polydispersed in size. No direct information on the particle size could be obtained from the small angle scattering measurements because the lower limit of the q range was too high for such analysis.

### 3.4. Evaluation of the Transfection Efficacy of Hybrid Particles In Vitro

**Figure 5:**
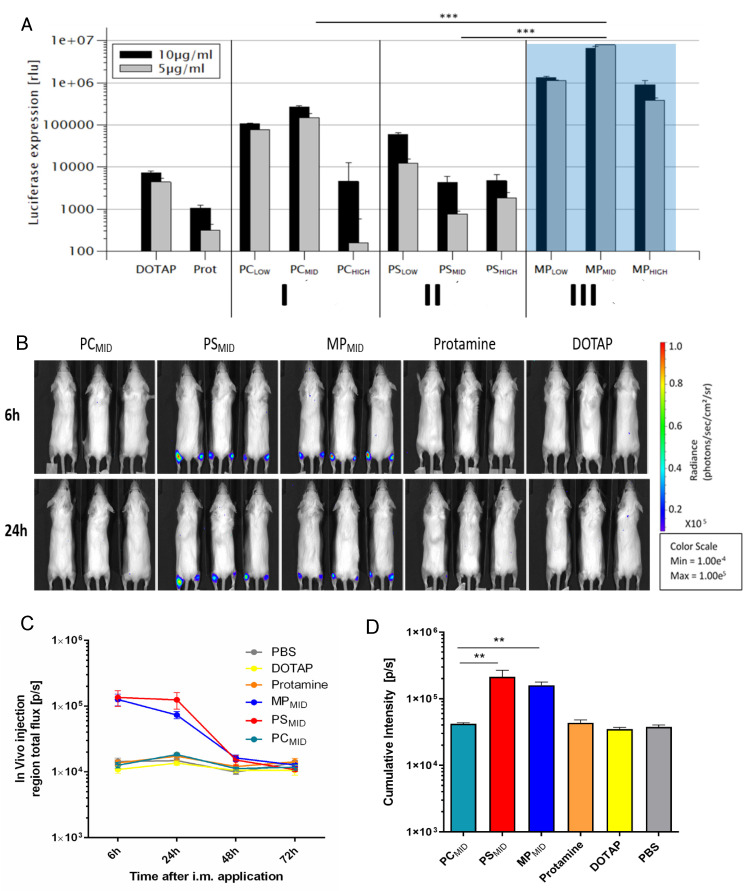
(**A**) HEK293 cell transfection of different protamine/DOTAP hybrid nanoparticles at different compositions in comparison to protamine/mRNA and DOTAP/mRNA particles. *** *p* < 0.001. Statistical significance was determined using two-way ANOVA combined with a Bonferroni comparison test. (**B**) Bioluminescence imaging of mice (*n* = 3 for particles, *n* = 2 for negative control) 6, 24, and 48 h after i.m. injection in the footpad of PC_MID_, PS_MID_, MP_MID_, protamine/mRNA, DOTAP/mRNA or only PBS buffer (not shown). The average radiance is quantified as photons/sec/cm2/sr, meaning the amount of photons that are emitted per second per square centimeter tissue per square radian (solid angle). (**C**) Total flux kinetic analysis of the samples with respect to time. (**D**) Cumulative bioluminescence signal intensity at all time points indicating protein expression following injection of the respective samples. ** *p* < 0.01. Statistical analysis was performed using one-way ANOVA with Tukey’s multiple comparisons test.

The effectiveness of the mRNA transfection for the different delivery systems as described above was investigated in vitro and in vivo by using luciferase-coding mRNA (Figure 5). Particles comprised only one of the two excipients: DOTAP/mRNA and protamine/mRNA were compared to the hybrid protamine/DOTAP/mRNA particles with the three topologies. The in vitro transfection efficiency (Figure 5A) of the systems with only one excipient, DOTAP/mRNA and protamine/mRNA, was only moderate and low, respectively, compared to the hybrid particles. The combination of two complexing agents was beneficial to achieve higher cell transfection at equivalent mRNA doses. The sequential assembly of a negatively charged polymer core (PC_LOW_ and PC_MID_) with a cationic lipid led to an increase of the transfection by a factor of 10–100. The cell transfection of the hybrid systems was higher when DOTAP was added to the protamine/RNA core particles (PC particles) compared to particles with the DOTAP/RNA core to which protamine was added (PS particles). Therefore, the above described differences in the particle morphology with the same overall composition were mirrored by differences in the transfection efficiency. The highest transfection efficiency was observed when using the single-step protocol for nanoparticle formation (MP) in which DOTAP and protamine were combined prior to mRNA addition. Here, at a composition of 45% protamine and 55% DOTAP (mol% positive charge, MP_MID_), signals were highest. For this protocol, an internal composite organization consisting of small lamellar patches embedded in a homogenous matrix without detectable ordering was observed (Figure 5C). It may be hypothesized that this composite organization was related to the synergistic effects between protamine and DOTAP complexation for mRNA transfection.

For most particles, the cell viability was about 80% compared to the control (Figure S8). Only MP particles showed mild toxic effects at high concentrations (60% cell viability at 10 µg/mL). The same particles were tested in C2C12 cell lines with comparable superiority of MP particles and no toxic effects (Figures S9 and S10).

### 3.5. Evaluation of the Transfection Efficacy of Hybrid Particles In Vivo

The different types of hybrid particles were tested for their efficacy in vivo after intramuscular application. Mice were treated with either buffer, particles composed of mRNA and one complexing agent (protamine/mRNA and DOTAP/mRNA) or both complexing agents in the three different topologies (PC, PS, MP) at very similar qualitative and quantitative compositions (Figure 5B). Negative control, protamine/mRNA, and DOTAP/mRNA particles showed no transfection in vivo. Also, particles with a protamine/mRNA core and DOTAP added on top (PC) did not yield significant signals, even though in the in vitro studies these formulations showed superior transfection in comparison to those with the opposite assembly sequence (PS). A preference of HEK293 cells for DOTAP uptake, as reported in the literature, could be related to higher signals in vitro [68]. Due to the diversity of cells present in vivo, the HEK293 cell’s preference for DOTAP in terms of uptake does not translate in vivo. Rather, it seems that the release of the mRNA, which is dependent on the particle structure, has the relevant impact on the transfection efficacy in vivo.

Best in vivo transfection was observed for MP_MID_ and PS_MID_ particles. The transfection kinetics for both particle systems showed highest activity 6 h after administration and took place mainly at the injection site (Figure 5C). The cumulative transfection observed as bioluminescence over different time points was significantly higher for PS_MID_ and MP_MID_ compared to PC_MID_ (Figure 5D).

In summary, similar to the in vitro results, the in vivo combination of both complexing agents proved to be more effective than particles with only one complexing agent. Particles with the same composition can have a different internal organization and can display different efficiencies in transfection only due to their assembly sequence. PS_MID_ and MP_MID_ showed greater effects compared to PC_MID_ particles.

## 4. Discussion

Here, we have studied the impact of the assembly protocols as well as the impact of the portion of the components on the molecular organization and the transfection efficacy of hybrid polymer and lipid nanoparticles comprising mRNA as a therapeutic agent. The utilization of DOTAP and protamine as model systems and systematic variation of fundamental process steps allowed us to derive insight into the correlation of both structural characteristics as well as biological activity with the manufacturing scheme. Although being done with standardized model components, certain fundamental observations may be applicable to other similar hybrid systems. Since the particles are built up via the rather rigid mRNA phosphate backbone, transferability to other mRNAs with a comparable length is assumed. A transferability to higher deviations in the length of the RNA chain was not investigated.

The combination of protamine, DOTAP, and mRNA resulted in hybrid nanoparticles in which the particle topology was controlled by assembly protocols. Despite very similar overall particle compositions, differences were observed not only in physicochemical properties such as size, zeta potential, internal particle organization, and accessible mRNA, but also in mRNA release and transfection efficiency in vitro and in vivo.

A peak in the SAXS and SANS curves was assigned to the typical lipid bilayer structure with intercalated RNA as described previously [39,42,44,45]. This Bragg peak changed in area, but not with respect to the maximum position, depending on the manufacturing protocols. It was taken as a measure for the composite organization of the particles and gave insight into the competing interactions of DOTAP and protamine under the respective conditions. The peak was observed, for example, for negatively charged DOTAP/RNA complexes as precursors for the final PS particles, where, after protamine addition, its area decreased in correlation with the protamine to DOTAP ratio. Therefore, the protamine must have affected the pre-existing lipoplex organization, and the resulting structure was supposedly more complex than a plain core-shell geometry. Although the data do not allow us to prove that the protamine/RNA core particle structure was affected by the added DOTAP, since no Bragg peak was visible, it may be hypothesized that such interference can occur for the latter system.

The corresponding cryo-TEM picture confirmed the presence of moieties with lamellar organization within different particle types. Although, in comparison to SAXS and SANS, the method is less quantitative, it allowed us to reveal certain structural characteristics, which were specific to the systems assembled directly from the mixed DOTAP and protamine together. A composite organization, with patches of a small region with lamellar organization, dispersed in a less structured, dense phase was found characteristic for this assembly route. With these characteristics, the systems described here are different from other, earlier described polymer-lipid hybrid particles of a wide variety of materials manufactured from classical methods, for which a polymer-core-lipid-shell structure was described or assumed [17,18,19,20,21].

With respect to the biological activity, the hybrid particles, obtained from the different ways of combination of the two complexing agents, showed a significantly higher transfection compared to the particles with only one of the two complexing agents. The superior efficacy confirms our expectations as this was also observed for different lipid-polymer hybrid nanoparticles by other groups [20,69]. With the experiments presented here, the different transfection signals of mRNA within the hybrid nanoparticles could be directly correlated with different structural morphologies obtained from varying the assembly procedure and the component composition. The strongest improvement in biological activity was observed for particles manufactured directly from the DOTAP/protamine mixture (MP), which were characterized by the composite organization of the lamellar patches inside the unstructured matrix. Also, the PS particles, where protamine is added to preexisting DOTAP/RNA lipoplexes and interferes with the lamellar organization, showed good efficacy. Apparently, a similar molecular organization to the MP particles was achieved, which could not be obtained with the opposite assembly sequence. The different binding strengths within the above-mentioned competing interactions can be considered to be the foundation for these observations.

These findings were corroborated by the results from further physicochemical characterization, including size and zeta potential measurements and heparin release assay. The heparin-induced mRNA release varied strongly according to the preparation protocol and thus, particle morphology. The release was relatively low for sequentially assembled particles (PS, PC, with PC being lower than PS) and high for the single-step assembly (MP), indicating higher accessibility of the cationic groups to heparin and thus higher mRNA release. The facilitated mRNA release can therefore be one explanation for a higher in vivo transfection. However, due to the complexity of living organisms, a simple release assay is scarcely translatable to in vivo results, thus a variety of ex vivo experiments is necessary for any prediction in terms of the cell transfection efficiency of mRNA particles.

In addition, the Quant-iT RiboGreen assay (Figure 2 and Table S1, right column) showed twice the amount of accessible mRNA when the cationic complexation agents are mixed together prior to mRNA addition (MP).

In summary, the results demonstrate that hybrid particles may allow to substantially improve transfection efficacy. For these complex systems, the control of attributes which are important for product quality and the understanding of structure function coherencies are of fundamental importance. In this report it was shown that the order of assembling composite nanometer-sized pharmaceuticals and their resulting molecular organization are important for the biological activity. Advanced characterization such as by SANS, SAXS, and cryo-TEM was used as a tool to gain insight into the relationship between the conditions of production, the intrinsic molecular organization of mRNA delivery vehicles, and their biological activity. The in vivo proof for these options to modulate RNA expression was given by intramuscular injection of RNA nanoparticles in mice. Such understanding can provide a basis to facilitate future developments of complex RNA delivery systems with tailored characteristics for a given therapeutic intervention such as in cancer therapy or in next-generation vaccine development.

## 5. Conclusions

Composite biopolymer and lipid nanoparticles carrying mRNA were manufactured by systematic variation of the assembly route and proportion of materials. The different products were investigated regarding their molecular organization and biological function. A relationship between the molecular organization derived from SANS, SAXS, and cryo-TEM measurements, together with the mRNA release, and the functional transfection capacity of the particles in vitro and in vivo was observed. The combination of the two complexing agents, cationic lipid and cationic polymer, resulted in improved transfection of mRNA in comparison to the single complexing agents. Here, the single-step protocol, where the complexing agents were mixed together prior to mRNA addition, led to mixed hybrid nanoparticles that showed an inhomogeneous internal organization and a higher transfection efficacy in comparison to the sequentially assembled particles. This may be due to the self-assembly of the complexing agents resulting in a facilitated release of mRNA as well as differences in the structural morphology and the surface composition. The results underline that by modifying the manufacturing procedure, the structural characteristics as well as the transfection capacity of the particles can be modulated. The improved efficacy of this type of composite materials, where low ordered domain materials with different properties are combined, may be a concept that can be realized by using a wider variety of different materials for nanoparticle formation.

## Figures and Tables

**Table 1:** Physicochemical characterization of the final particles from the three different assembly sequences (MP, PS, PC), each with three different protamine contributions to the N/P ratio composition (LOW, MID, HIGH).

Name	Z_ave_ [nm]	PDI	ZP [mV]	mRNA [%]
MP_LOW_	217 ± 41	0.21 ± 0.06	47 ± 18	9.8 ± 5.5
MP_MID_	182 ± 27	0.20 ± 0.04	44 ± 12	9.8 ± 5.4
MP_HIGH_	147 ± 38	0.26 ± 0.13	34 ± 17	6.8 ± 4.1
PS_LOW_	166 ± 10	0.25 ± 0.04	38 ± 24	4.6 ± 1.8
PS_MID_	202 ± 46	0.26 ± 0.11	28 ± 14	4.3 ± 0.7
PS_HIGH_	234 ± 75	0.26 ± 0.07	27 ± 09	5.0 ± 1.8
PC_LOW_	148 ± 48	0.21 ± 0.03	48 ± 27	3.8 ± 1.6
PC_MID_	146 ± 21	0.19 ± 0.02	39 ± 19	3.3 ± 1.8
PC_HIGH_	160 ± 43	0.18 ± 0.04	35 ± 20	4.0 ± 1.0

Mean particle size (Z_ave_), polydispersity index (PDI), zeta potential (ZP), and the percentage of accessible mRNA (mRNA) were obtained from DLS/ELS and Quant-iT RiboGreen measurements. For detailed information on the data, see Table S2 (supplement). Each data point resembles the mean value of at least three independent samples measured in triplicate.

## Supplementary Materials

The following are available online at https://www.mdpi.com/2073-4409/9/9/2034/s1, Figure S1: Contrast matching analysis of protamine (PDF), Figure S2: Contrast matching analysis of DOTAP (PDF), Figure S3: Contrast matching analysis of RNA (PDF), Figure S4: Contrast matching analysis of PS_MID_ (PDF), Figure S5: Contrast matching analysis of PC_LOW_ (PDF), Figure S6: Contrast matching analysis of PS_HIGH_ (PDF), Figure S7: Contrast matching analysis of PC_LOW_ peak (PDF); Figure S8: HEK293 cell viability (PDF), Figure S9: C2C12 cell transfection (PDF), Figure S10: C2C12 cell viability (PDF), Table S1: Manufacturing details (PDF), Table S2: Physicochemical analysis details (PDF), Table S3: Theoretical and measured contrast matching points of the single components (PDF).

## Patents

A patent application has been submitted by BioNTech and the Johannes Gutenberg University including the following authors as inventors: Christian D. Siewert, Heinrich Haas, Vera Cornet, Sara S. Nogueira and Peter Langguth.

## Abbreviations

mRNA	messenger RNA
SANS	small angle neutron scattering
cryo-TEM	cryogenic transmission electron microscopy
DOTAP	1,2-Dioleoyl-3-trimethylammonium-propane
RNAse	ribonuclease
LNP	lipid nanoparticle
DLS	dynamic light scattering
ELS	elastic light scattering
D-contrast	Deuterium contrast
HEPES	2-[4-(2-hydroxyethyl) piperazin-1-yl] ethane sulfonic acid
FBS	fetal bovine serum
PC	polymer core
PS	polymer shell
MP	mixed particle
TE	Tris-EDTA
HBG	hepes buffered glucose
JCNS	Jülich Centre for Neutron Science
MLZ	Heinz Maier-Leibnitz Zentrum
NSLD	neutron scattering length density
DESY	Deutsches Elektronen Synchroton
ANOVA	analysis of variance
i.m.	intramuscular
