# Supplementary material for: Hybrid Biopolymer and Lipid Nanoparticles with Improved Transfection Efficacy for mRNA
Source: Cells. 2020 Sep 5;9(9):2034. doi: 10.3390/cells9092034 (PMC7563888; doi:10.3390/cells9092034)
Supplement: Supplementary file 1 [file cells-09-02034-s001.pdf]

Supplementary data of: Hybrid biopolymer and lipid nanoparticles display improved transfection efficacy for mRNA

Christian D. Siewert [1], Heinrich Haas [2], Vera Cornet [1], Sara S. Nogueira [2], Thomas Nawroth [1], Lukas C. J. Uebbing [1], Antje Ziller [1], Jozef Al-Gousous [1], Aurel Radulescu [3], Martin A. Schroer [4], Clement E. Blanchet [4], Dmitri I. Svergun [4], Markus P. Radsak [5], Ugur Sahin [2,6] , Peter Langguth[1,*]

[1] Department of Pharmaceutics and Biopharmaceutics, Johannes Gutenberg University Mainz, D-55131 Mainz, Germany
[2] BioNTech RNA Pharmaceuticals, D-55131 Mainz, Germany
[3] Jülich Centre for Neutron Science JCNS at Heinz Maier-Leibnitz Centrum MLZ, D-85748 Garching, Germany
[4] European Molecular Biology Laboratory EMBL Hamburg Outstation c/o Deutsches Elektronen Synchrotron DESY, 22603 Hamburg, Germany
[5] IIIrd Dept. of Medicine, Johannes Gutenberg University Medical Center, Johannes Gutenberg University, D-55131 Mainz, Germany
[6] Exp. Oncology, IIIrd Dept. of Medicine, TRON, Johannes Gutenberg University Medical Center, Johannes Gutenberg University, D-55131 Mainz, Germany
* Correspondence: langguth@uni-mainz.de Tel: +49-6131- 3925746. Fax: +49-6131/39 – 25021

Table s1. Preparation of hybrid lipid/polymer particles varying the N/P ratio and the manufacturing method. The sum of the N/P ratio is equal to 2. The weight ratio is given in percent Protamine:DOTAP:RNA (P:D:R.)

	Name/Remark	Overall N/P ratio: 2		Conc. Protamine	Conc. DOTAP	Conc. RNA	Weight ratio (P:D:R)
		N/P ratio Protamine	N/P ratio DOTAP	[mg/ml]	[mg/ml]	[mg/ml]	[%]
	DOTAP/mRNA (0%)	0	2	0	1	0.25	(0/80/20)
I	PC_{LOW} (15%)	0.3	1.7	0.06	0.85	0.25	(5/73/22)
	PC_{MID} (45%)	0.9	1.1	0.18	0.55	0.25	(18/56/26)
	PC_{HIGH} (85%)	1.7	0.3	0.35	0.15	0.25	(47/20/33)
II	PS_{LOW} (15%)	0.3	1.7	0.6	0.85	0.25	(5/73/22)
	PS_{MID} (55%)	1.1	0.9	0.22	0.44	0.25	(24/48/27)
	PS_{HIGH} (85%)	1.7	0.3	0.35	0.15	0.25	(47/20/33)
III	MP_{LOW} (15%)	0.3	1.7	0.06	0.85	0.25	(5/73/22)
	MP_{MID} (45%)	0.9	1.1	0.18	0.55	0.25	(18/56/26)
	MP_{HIGH} (85%)	1.7	0.3	0.35	0.15	0.25	(47/20/33)
	Protamine/mRNA (100%)	2	0	0.41	0.00	0.25	(62/0/38)

Table s2. Physicochemical evaluation / characterization results from the three different systems, each with three different Protamine concentrations. ZAverage, PDI and Zeta potential was obtained from DLS/ELS. Free mRNA in H2O and FBS was obtained from the Quant-iT™ Ribogreen® RNA reagent kit. Data are shown as mean ± S.D. for each experiment.

Name	Protamine contribution to overall positive charge [%]	$Z_{Average}$ [nm]	PDI	Zeta Pot. [mV]	Accessible mRNA in TE-buffer [%]
DOTAP/mRNA	0	154±13	0.23±0.005	67±4	6.3±4
PC$_{LOW}$	15	148±48	0.21±0.06	48±27	3.8±1.6
PC$_{MID}$	45	146±21	0.20±0.04	39±19	3.3±1.8
PC$_{HIGH}$	85	160±43	0.26±0.13	35±20	4±1
PS$_{LOW}$	15	166±10	0.25±0.04	38±24	4.6±1.8
PS$_{MID}$	55	202±46	0.26±0.11	28±14	4.3±0.7
PS$_{HIGH}$	85	234±75	0.26±0.07	27±9	5±1.8
MP$_{LOW}$	15	217±41	0.21±0.03	47±18	9.8±5.5
MP$_{MID}$	45	182±27	0.19±0.02	44±12	9.8±5.4
MP$_{HIGH}$	85	147±38	0.18±0.04	34±17	6.8±4.1
Protamine/mRNA	100	305±47	0.31±0.06	29±6	5.7±4.4
PC$_{MID}$ intermediate		*151±35*	*0.15±0.05*	*-24±5*	*n.a.*
PS$_{MID}$ intermediate		*188±5*	*0.31±0.04*	*-14±8*	*n.a.*
MP$_{MID}$ intermediate		*211±39*	*0.36±0.02*	*79±3*	*n.a.*

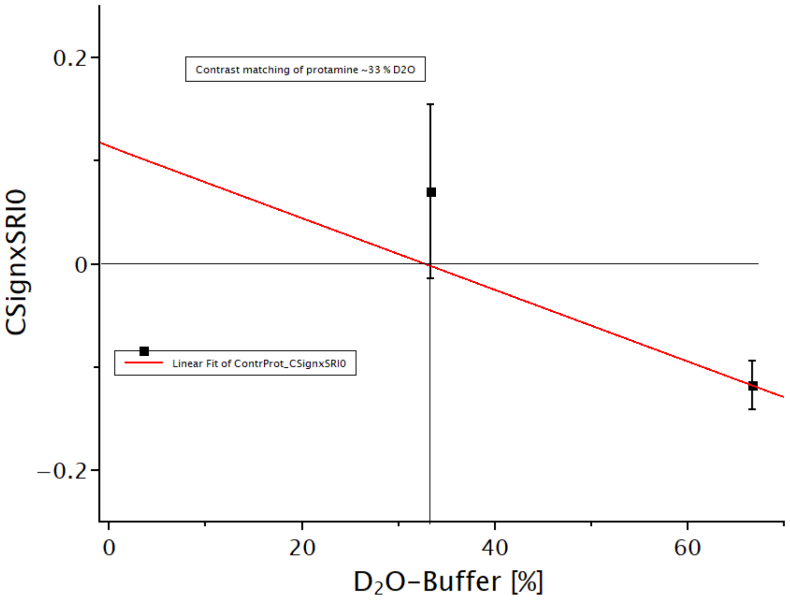

Figure s1. Contrast matching point analysis. I_0 was obtained by extrapolating the scattering curve at low q values. The square root of I_0 plotted against the D_2O concentration in buffer yield the D_2O matching point and scattering length densities of protamine.

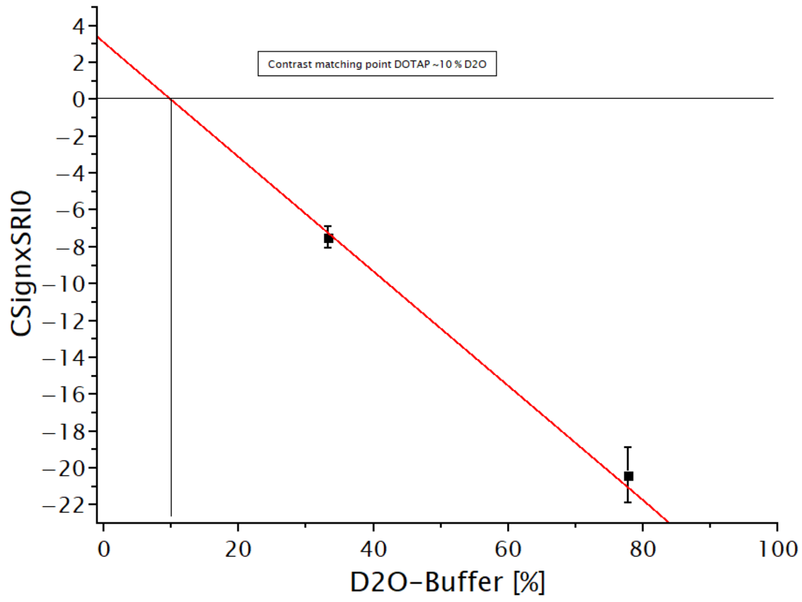

Figure s2. Contrast matching point analysis. I_0 was obtained by extrapolating the scattering curve at low q values. The square root of I_0 plotted against the D_2O concentration in buffer yield the D_2O matching point and scattering length densities of DOTAP.

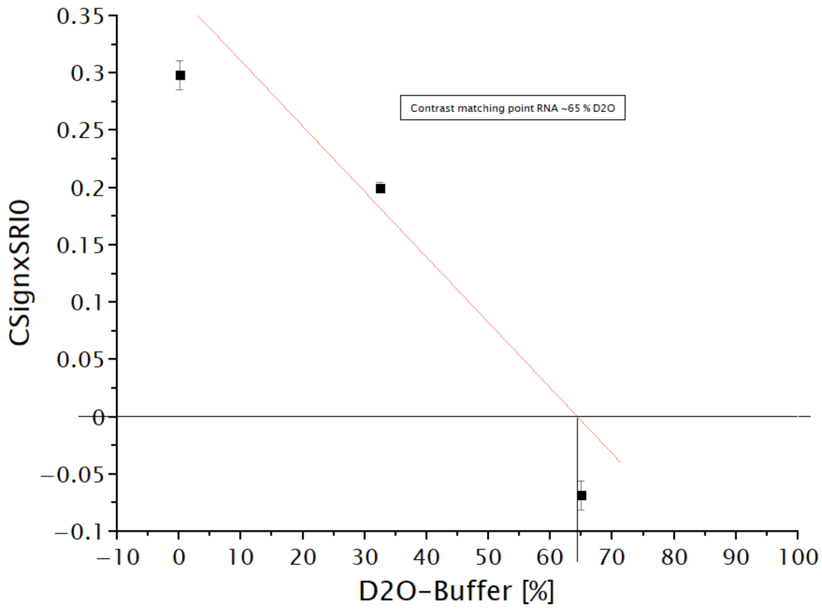

Figure s3. Contrast matching point analysis. I_0 was obtained by extrapolating the scattering curve at low q values. The square root of I_0 plotted against the D_2O concentration in buffer yield the D_2O matching point and scattering length densities of RNA.

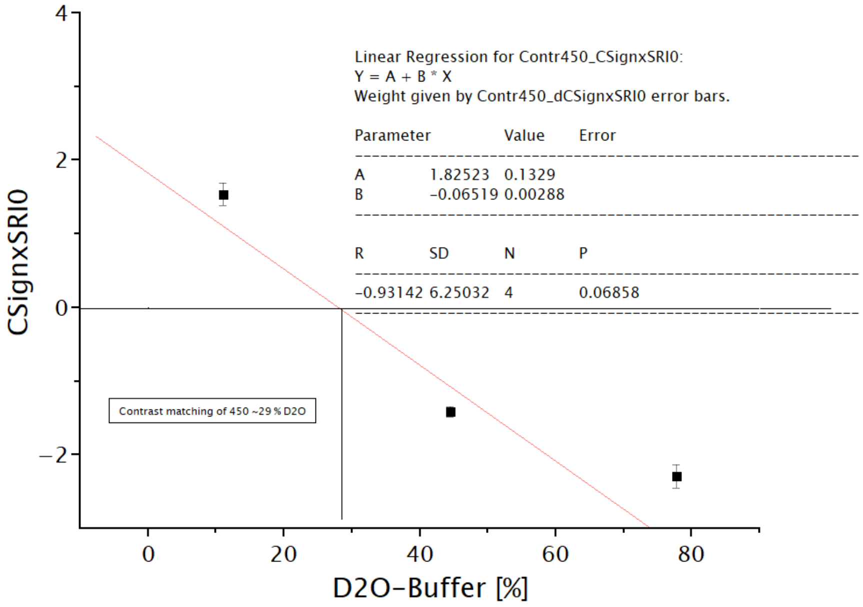

Figure s4. Contrast matching point analysis. I_0 was obtained by extrapolating the scattering curve at low q values. The square root of I_0 plotted against the D_2O concentration in buffer yield the D_2O matching point and scattering length densities of PSMID.

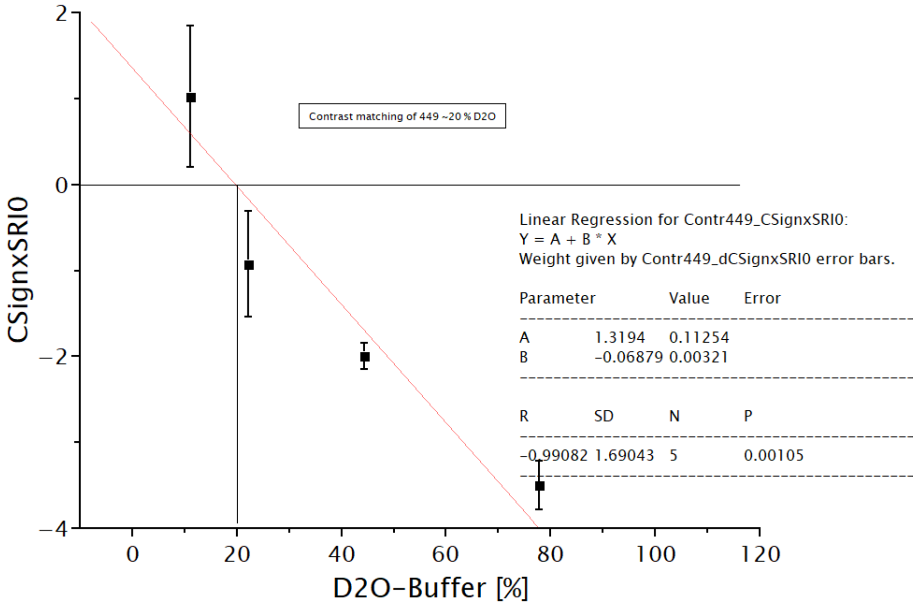

Figure s5. Contrast matching point analysis. I_0 was obtained by extrapolating the scattering curve at low q values. The square root of I_0 plotted against the D_2O concentration in buffer yield the D2O matching point and scattering length densities of PCLOW.

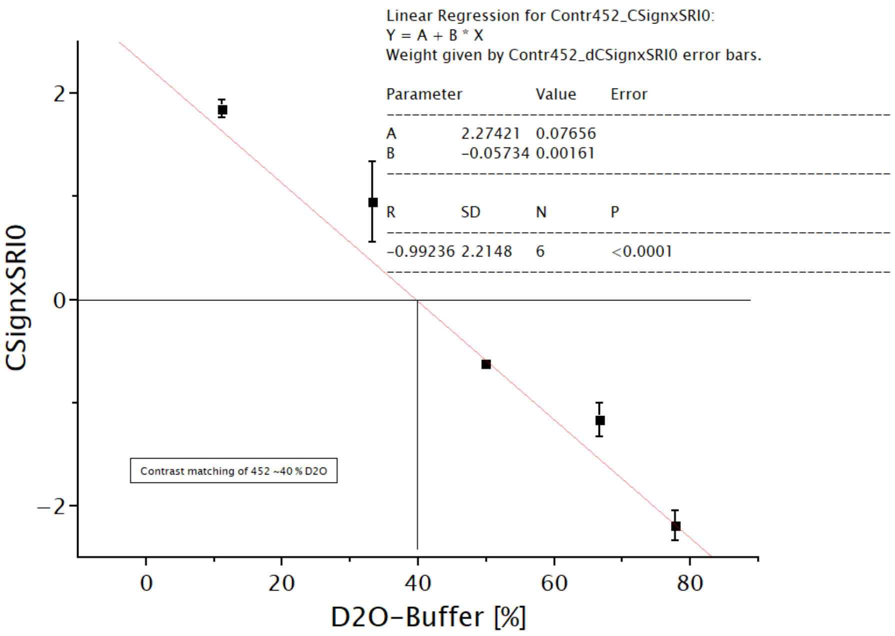

Figure s6. Contrast matching point analysis. I_0 was obtained by extrapolating the scattering curve at low q values. The square root of I_0 plotted against the D_2O concentration in buffer yield the D_2O matching point and scattering length densities of PS_{HIGH}.

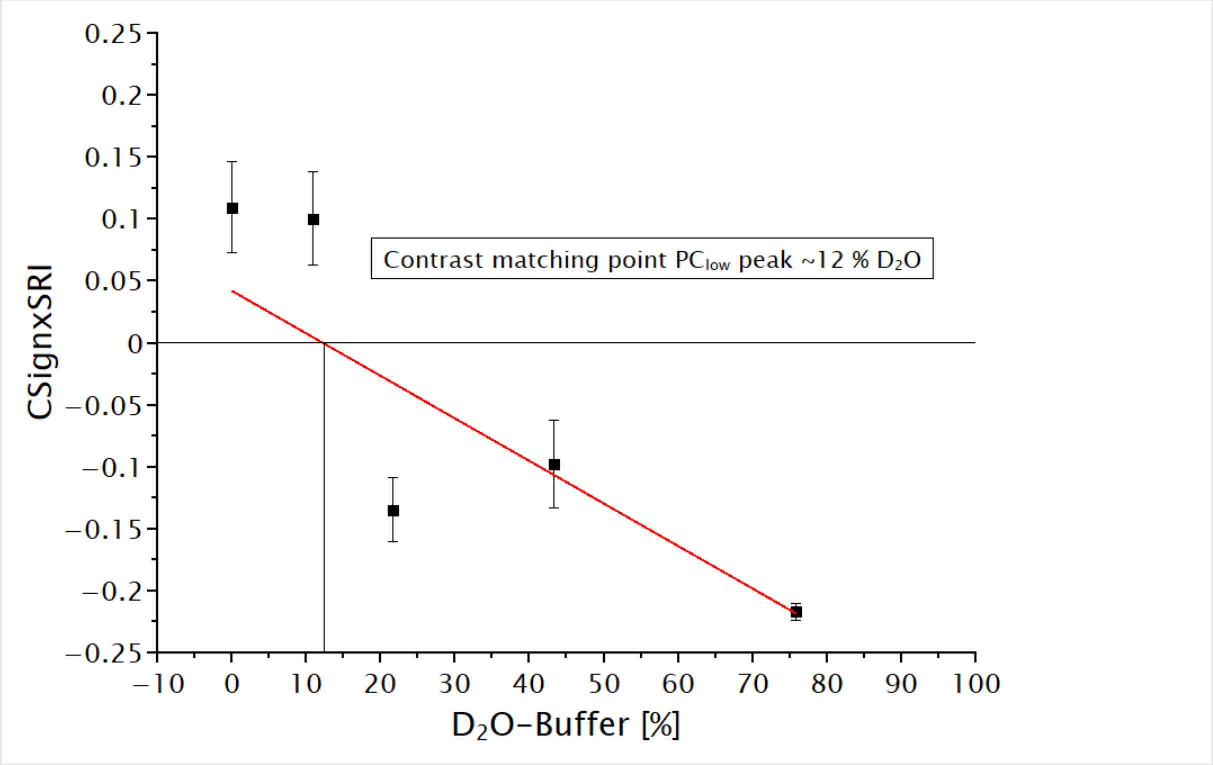

Figure s7. CMP analysis of the peak at a q-range from 0.077-0.14 Å^{-1}. √(Σ(I(sample) - I(blank))) was plotted against the D_2O concentration in buffer to yield the D_2O matching point and scattering length densities of the PC_{LOW} peak.

For DOTAP the contrast matching is approx. ~10 %, for protamine ~36 % and for mRNA ~67 %. Considering the mass ratio and the theoretical contrast matching values from the single components, a theoretical D_2O amount in medium can be estimated for which the scattering of the particles is matched. In

general, all obtained contrast matching points are comparably lower than those expected, indicating a systemic error in the results. Nonspecific aggregation results in an increase at low q values and overestimation of both ,radius of gyration (R_g) and I(0), and therefore can result in lower overall contrast matching points [1]

Table s3: Theoretical and measured contrast matching points of the single components and the respective particle systems [2,3].

Sample	Weight ratio (P:D:R) [%]	Estimated D_2O matching point[%]	Obtained D_2O contrast matching point [%]
DOTAP	(0:100:0)	10-15 [3]	10
Protamine	(100:0:0)	45-50 [3]	36
mRNA	(0:0:100)	65-70 [3]	65
PC_{LOW}	(5/73/22)	22-26	20
PS_{MID}	(24/48/27)	29-33	29
PS_{HIGH}	(47/20/33)	42-46	40

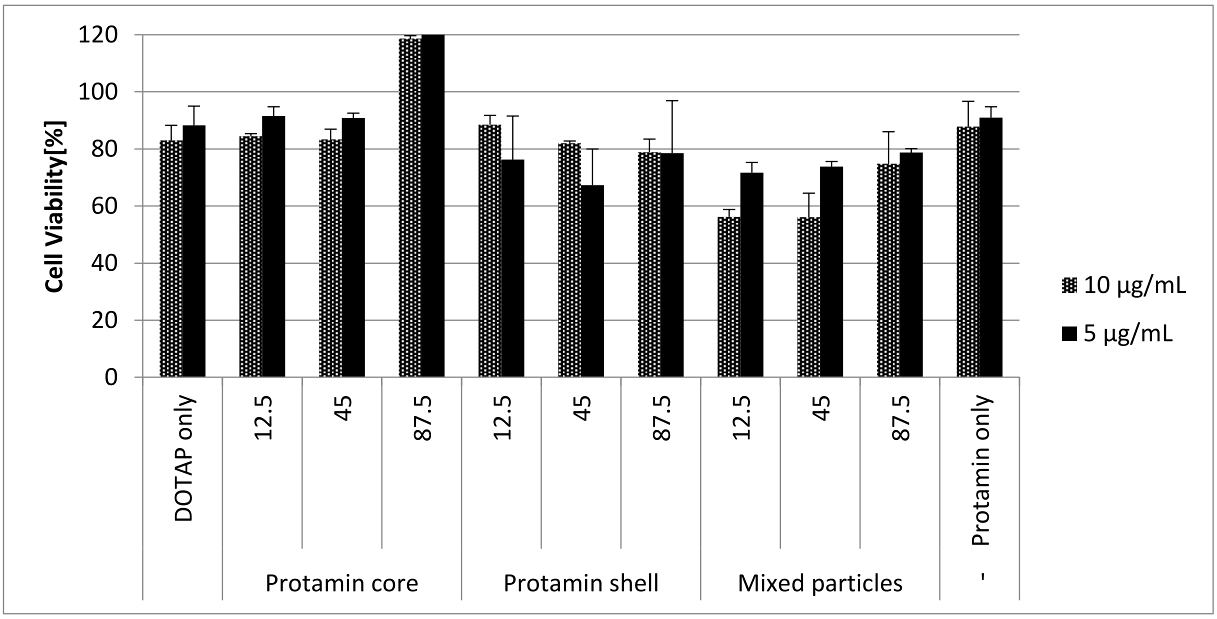

Figure s8. HEK293 cell viability of Protamine/DOTAP hybrid core/shell nanoparticles at different concentrations in comparison to sole Protamine/mRNA and DOTAP/mRNA particles.

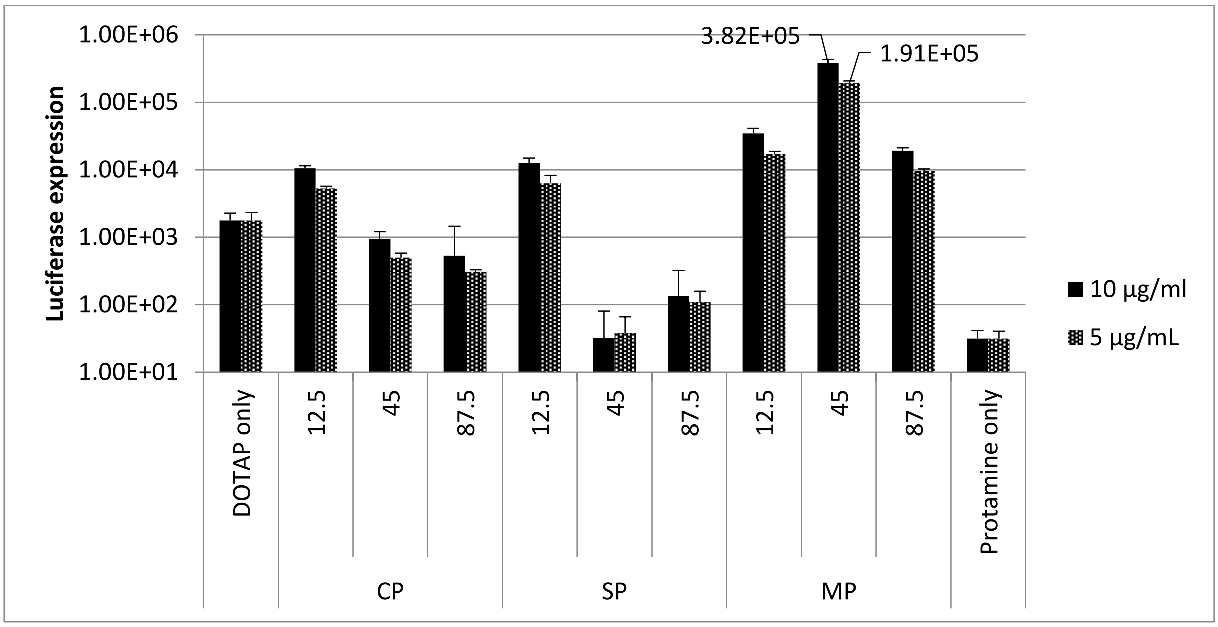

Figure s9. C2C12 cell transfection of different Protamine/DOTAP hybrid core/shell nanoparticles at different concentrations in comparison to sole Protamine/mRNA and DOTAP/mRNA particles.

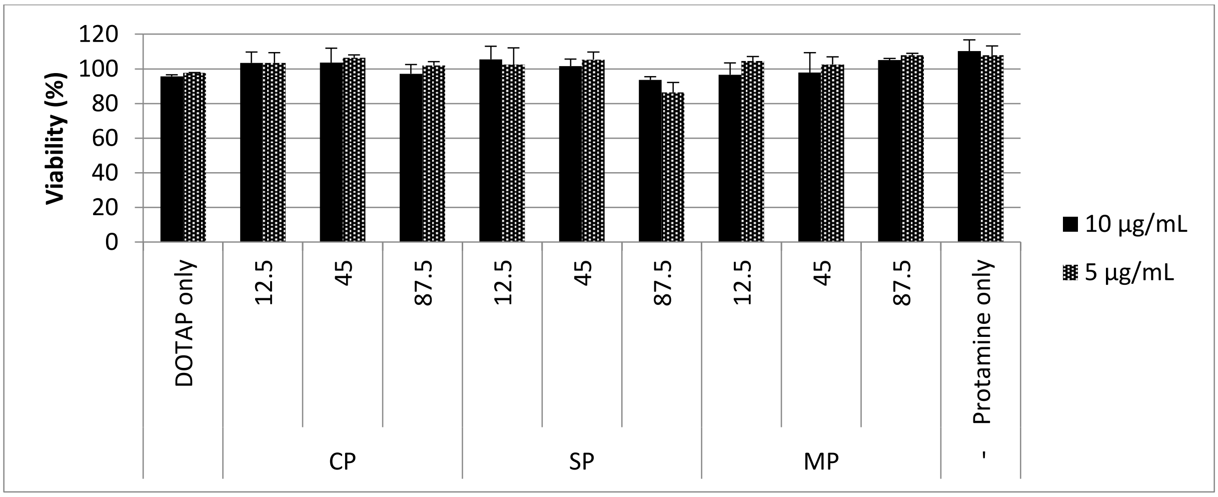

Figure s10. C2C12 cell viability of Protamine/DOTAP hybrid core/shell nanoparticles at different concentrations in comparison to sole Protamine/mRNA and DOTAP/mRNA particles.

1 Blanchet, C.E., Svergun, D.I., Small-Angle X-Ray Scattering on Biological Macromolecules and Nanocomposites in Solution, *Annu. Rev. Phys. Chem*. **2013**, *64*, 37–54. https://doi.org/10.1146/annurev-physchem-040412-110132.

2 Sears, V.F., Neutron News Neutron scattering lengths and cross sections, *Neutron News*. **1992**, *3:3*, 26–37. https://doi.org/10.1080/10448639208218770.

3 Ashkar, R., Bilheux, H.Z., Bordallo, H., Briber, R., Callaway, D.J.E., Cheng, X., Chu, X.-Q., Curtis, J.E., Dadmun, M., Fenimore, P., Fushman, D., Gabel, F., Gupta, K., Herberle, F., Heinrich, F., Hong, L., Katsaras, J., Kelman, Z., Kharlampieva, E., Kneller, G.R., Kovalevsky, A., Krueger, S., Langan, P., Lieberman, R., Liu, Y., Losche, M., Lyman, E., Mao, Y., Marino, J., Mattos, C., Meilleur, F., Moody, P., Nickels, J.D., O'dell, W.B., O'neill, H., Perez-Salas, U., Peters, J., Petridis, L., Sokolov, A.P., Stanley, C., Wagner, N., Weinrich, M., Weiss, K., Wymore, T., Zhang, Y., Smith, J.C., Neutron scattering in the biological sciences: progress and prospects, *Acta Cryst*. **2018**, 1129–1168. https://doi.org/10.1107/S2059798318017503.
